# High variability phonetic training in adaptive adverse conditions is rapid, effective, and sustained

**DOI:** 10.1371/journal.pone.0204888

**Published:** 2018-10-09

**Authors:** Christine Xiang Ru Leong, Jessica M. Price, Nicola J. Pitchford, Walter J. B. van Heuven

**Affiliations:** 1 School of Psychology, University of Nottingham Malaysia Campus, Semenyih, Selangor, Malaysia; 2 School of Psychology, University of Nottingham, Nottingham, United Kingdom; Baycrest Health Sciences, CANADA

## Abstract

This paper evaluates a novel high variability phonetic training paradigm that involves presenting spoken words in adverse conditions. The effectiveness, generalizability, and longevity of this high variability phonetic training in adverse conditions was evaluated using English phoneme contrasts in three experiments with Malaysian multilinguals. Adverse conditions were created by presenting spoken words against background multi-talker babble. In Experiment 1, the adverse condition level was set at a fixed level throughout the training and in Experiment 2 the adverse condition level was determined for each participant before training using an adaptive staircase procedure. To explore the effectiveness and sustainability of the training, phonemic discrimination ability was assessed before and immediately after training (Experiments 1 and 2) and 6 months after training (Experiment 3). Generalization of training was evaluated within and across phonemic contrasts using trained and untrained stimuli. Results revealed significant perceptual improvements after just three 20-minute training sessions and these improvements were maintained after 6 months. The training benefits also generalized from trained to untrained stimuli. Crucially, perceptual improvements were significantly larger when the adverse conditions were adapted before each training session than when it was set at a fixed level. As the training improvements observed here are markedly larger than those reported in the literature, this indicates that the individualized phonetic training regime in adaptive adverse conditions (HVPT-AAC) is highly effective at improving speech perception.

## Introduction

Non-native English speakers have often difficulties discriminating phonemic contrasts that involve phoneme categories that are not present in their native language. For example, Japanese speakers have difficulties distinguishing between the English phonemes /r/ and /l/ [1–4], Mandarin speakers find it difficult to discriminate between the English voiced-unvoiced stops /t/ and /d/ in word-final positions [5, 6], and Spanish speakers have difficulties with the English vowels /æ/ and /ʌ/ [5]. These difficulties are explained by a number of theories and models of non-native speech perception. According to Best’s Perceptual Assimilation Model [7], difficulties arise when phonemes from the non-native language are assimilated inappropriately into native language phonetic categories. Kuhl’s [8] Native Language Magnet Theory proposes that native speech sound categories serve as magnets that increase perceived similarity of the phonetic sounds close to the phonetic representations. This perceptual magnet effect causes difficulties for non-native speakers to discriminate acoustically similar, but linguistically distinctive non-native phonetics sounds. According to Flege’s Speech Learning Model [9], the discrimination difficulty of non-native speakers prevents the formation of appropriate phonetic categories to represent the non-native phonetic sounds. Therefore, phonemic pairs are typically assimilated into one native category by the non-native speakers and overcoming the native language interference may be critical to successfully master these non-native distinctions.

A large number of studies have investigated perceptual phonetic training methods to improve speech perception in the non-native language using either synthetic (e.g., [10–12]) or natural (e.g., [12–14]) speech stimuli. The most common task used in perceptual training studies is the identification task, in which participants determine the identity of the auditory stimuli presented by deciding which of the written words presented on the screen matches the word heard and are provided with feedback after each response [1, 2, 4, 13–22]. However, some studies have used an identification task and a discrimination task in which participants determine whether auditory stimuli presented are identical or not [3, 5, 23–25]. An auditory discrimination task has the advantage that participants do not need to know how to read words in the language trained.

One of the most successful perceptual training methods was introduced by Lively and colleagues who showed the beneficial effects of training with highly variable material (later referred to as High Variability Phonetic Training or HVPT) with Japanese native speakers learning the English /r/-/l/ contrast [13–15]. A typical HVPT paradigm trains listeners with highly variable speech tokens which are usually produced by multiple speakers. Furthermore, target phonemes in this paradigm are presented in different phonetic contexts (i.e., at word initial, middle or final positions). The majority of studies in the literature have reported significant (weak to moderate) training benefits in perceptual phonetic training (e.g., [6, 13]), that lasted up to six months in some studies [15, 17]. The training benefits observed usually generalized to novel/untrained stimuli (of the same phonemic contrasts) that had not been used as training material, and to stimuli produced by novel speakers who had not been heard during training (e.g., [4, 19]). When participants in Lively et al. [14] were trained with single speaker stimuli (low variability), training did not generalize to novel stimuli and speakers because participants developed highly detailed representations of trained stimuli. Lively et al. [15] concluded therefore that high variability training materials promotes the generalization of the training. Although phonetic training benefits are well-established, training procedures used in the literature often take days if not weeks to be completed before improvements in phonetic perception is observed. Thus, it is important to find ways to improve the effectiveness of perceptual training.

A large number of studies have demonstrated that compared to native speakers, speech perception in adverse conditions is particularly challenging for non-native speakers (e.g., [26, 27]). Adverse conditions affect the intelligibility of the speech and they occur because of, for example, environmental or transmission degradation (for an overview, see [28]). Environmental degradation can be created by presenting speech in noise (e.g., white or pink noise that produce predominantly energetic masking) or by presenting the speech with background talkers (e.g., produce predominantly informational masking). The detrimental effect of energetic masking is greater on non-native speakers compared to native speakers in word discrimination tasks involving white noise [29] and with comprehending connected speech in sentences presented in pink noise (e.g., [30, 31], for a review, see [32]).

A series of studies conducted by Bradlow and colleagues showed that the non-native deficit in speech-in-noise perception may be attributed to their perceptual strategy that is different from the native speakers. An example of a native speakers’ strategic perceptual approach can be seen in the clear speech effect, which is the intelligibility difference between clear speech and normal conversational speech. In clear speech production, the acoustic salience of the speech signals is enhanced. Native speakers in the study by Bradlow and Bent [33] benefited more from clear speech production than non-native speakers when access to the target speech signals was impeded by background noise. Bradlow and Bent attributed this native speaker advantage to the strategic allocation of attention to language-specific acoustical cues that facilitate speech comprehension. Non-native speakers showed a smaller clear speech effect because they benefited only from the enhancement of the overall acoustic salience. The authors pointed out that extensive experience with the target language was crucial to develop a native perceptual strategy. Furthermore, non-native speakers also need greater signal clarity (e.g., with clear speech production) before other compensatory information (e.g., semantic cues to predict word identity in sentence) can be used for speech perception in noise [34]. Another disadvantage that non-native speakers have was revealed by Bent, Kewley-Port and Ferguson [35] who showed that non-native speakers are more affected by across-talker variation when identifying vowels in noise, even though the variation was within the normal range of native speakers.

When speech signals are degraded by an informational masker (e.g., multi-talker babble), non-native speakers’ difficulty in understanding speech can be attributed to the greater interference of the native phonetic system when there is ambiguity during second language (L2) processing (e.g., disrupted signal perceived due to the noisy environment), the increasing cognitive load in non-native speakers when processing two sources of non-native auditory information, or the non-native speakers’ inexperience in separating the signal from the masker language [32, 36], especially when they share similar spectrotemporal characteristics [37].

Although daily life conversations are often held in suboptimal or adverse conditions, very few phonetic training studies have been conducted with non-native normal-hearing listeners in difficult listening conditions. As far as we are aware, only Jamieson and Morosan [38] used background noise for some of the training materials to increase perceptual difficulty during the latter phase of their phonetic training; although no further attention was given to the implications of using background noise. In another related phonetic training study by Lengeris and Hazan [21], background noise was incorporated only in pre- and post-tests to examine the effect of training in quiet situations on non-native speech-in-noise perception. Furthermore, the Speech Perception Assessment and Training System for English as Second Language speakers (SPATS-ESL) developed by Miller and colleagues [39] trained English second language speakers with speech presented in multi-talker babble, but they focused on the syllable constituent perception and word identification in spoken sentences.

Training in noise, however, is common in perceptual training studies involving hearing-impaired listeners. In some of these studies, speech-shaped noise was used to simulate hearing loss in normal-hearing native listeners (e.g., [40]). A study by Burk and colleagues [41] with young normal-hearing and older hearing-impaired listeners showed that training in background noise improved word recognition performance in both groups, but their single talker training involved many hours (7 hours) and showed limited transfer from isolated presentation of trained words to presentation of these words in sentences.

Interestingly, novel research conducted by Hazan and colleagues (e.g., [42]) focused on the effects of audiovisual perceptual training. The study by Hazan, Kim and Chen [42] suggested that the reduction of information loaded on one channel (i.e., auditory) encouraged a heavier or more efficient weighting of another information processing channel (i.e., visual cues). Likewise, in adverse auditory training conditions where information from peripheral channels experiences interferences or is not available (i.e. no visual cues), participants are required to rely more on other cognitive processes to perform the perceptual task, such as a better selective attention strategy [28, 32]. Several studies have shown that the native speakers’ ability to identify and attend to critical language-specific acoustical cues that are more resistant to the adverse effects of noise, leads to the perceptual difference in speech-in-noise perception between native and non-native speakers [33–36]. Therefore, auditory training under adverse condition might help inexperienced non-native listeners to acquire these native-like selective attention strategies.

### The present study

The purpose of the present experiments was to examine the effectiveness of high variability phonetic training in adaptive adverse conditions (HVPT-AAC). The initial concept of HVPT-AAC was developed by the late Richard Pemberton, Kathy Conklin, Nicola Pitchford, and Walter van Heuven at the University of Nottingham. The implementation of HVPT-AAC for the research presented here was developed by the last two authors of this paper. HVPT-AAC uses natural speech stimuli (e.g., English minimal pairs) spoken by multiple speakers (e.g., with different English accents). Critical features of HVPT-AAC include presentation of spoken words in background noise in the form of multi-talker babble (adverse conditions) and an adaptive level of adverse conditions whereby an optimal training level can be set for each listener through an adaptive staircase procedure (see Experiment 2 for further details). Adverse conditions in perceptual training increases the speech perception difficulties not only for non-native speakers but also for highly proficient non-native speakers and native speakers whose disadvantage in speech perception may only manifests itself in the presence of noise (see [32] for a review). Studies incorporating background noise in perceptual training have found significant training effects (e.g., [38, 39, 41]). Therefore, HVPT-AAC is expected to improve speakers’ performance in perceiving phonetic distinctions.

HVPT-AAC involves a discrimination task in which participants determine whether the stimuli presented are identical or not. Spoken word identification and discrimination tasks involve different cognitive mechanisms. Identification training engages top-down processing of speech signals in which participants respond based on their categorized or phonetic representations in memory, whereas discrimination training influences primarily the bottom up processing of speech signals in which it engages lower level and sensory-based information in speech signals. Discrimination training improves speakers’ sensitivity to detect minor differences between similar sounding stimuli [11].

Flege’s Speech Learning Model [9] hypothesizes that the more dissimilar speech sounds are, the higher the chance is that they would be encoded into two distinctive phonological categories and identified as distinctive phonemic sounds. Similar to training studies that used discrimination training (more common in training studies that used synthetic training stimuli, e.g., [10–12]), HVPT-AAC training is designed to improve in particular participants’ sensitivity towards meaningful cues in non-native speech signals, to facilitate discrimination of between-category differences [43]. Perceptual sensitivity development was targeted based on the assumption that this ability reflects one of the native speakers’ advantages in native speech sound perception and that detection of such between-category differences is the fundamental limitation for non-native speakers. Another advantage of using a discrimination task is that it does not require prior knowledge of the training language and can be therefore ideal for novice language learners. For similar reasons, Giannakopoulou et al. [44] chose to use an oddity discrimination task to examine phonetic learning from their auditory word to pictures identification training. This discrimination task allowed them to test both real and nonsense words in young children, without orthographic interference. In the present study, discrimination improvements as a result of discrimination training are expected to facilitate the formation of higher-level linguistic representations of non-native phonetics; which can be assessed in the word identification task used in pre- and post-tests. Importantly, studies such as Handley, Sharples and Moore [3] and Shinohara and Iverson [45] support the use of a discrimination task in perceptual phonetic training, as the task was found to be as effective as the identification task.

To evaluate HVPT-AAC, the current experiments were conducted with moderately to highly proficient Malaysian English speakers receiving a university education delivered in English whilst resident in Malaysia. The three experiments reported below explore the effectiveness of HVPT conducted in adverse conditions (Experiment 1) and the effectiveness of adapting the level of adverse conditions in HVPT (HVPT-AAC) versus a fixed level of adverse conditions (Experiment 2). The longevity of training in adverse conditions were investigated in Experiment 3.

## Experiment 1

In this experiment we investigated whether perceptual training in two different levels of adverse conditions modulated the training results of non-native speakers, and whether the training generalized to untrained stimuli and untrained contrasts. Two levels of adverse conditions were created by manipulating the volume of the multi-talker babble (low vs. high) relative to the target stimulus volume level. We expected to see greater training benefits in the more adverse training condition (high volume) because listeners have to learn how to engage more effectively the cognitive processes used in the task (e.g., selective attention) when target auditory information is masked in the higher level of adverse conditions.

### Method

#### Participants

A group of 28 participants (aged 17–22; mean age 18.75; 16 females) who spoke Mandarin as their first language (L1) were recruited from the University of Nottingham, Malaysia Campus. All participants reported to have normal or corrected to normal vision, and they had no history of any hearing, speech, or reading problems. Participants were paid for their participation.

Participants completed a questionnaire to obtain information about their language background. Table 1 provides an overview of their mean age and their overall subjective proficiency scores for relevant languages (the overall scores were calculated by averaging reading, writing, speaking and listening ratings, scale: from 1 = very poor to 7 = fluent), as well as the age at which they first acquired Malaysian English (AoA).

**Table 1:** Participants’ demography, mean self-rated language proficiency in the two languages and English language test scores for the participants in the two adverse condition levels (low and high).

Adverse Conditions	N	Age	Self-rated English Proficiency	Self-rated Mandarin Proficiency	AoA	IELTS
Low	14	18.93	4.71	5.66	2.36	7.07
High	14	18.57	4.75	5.21	2.36	6.96

*Note*. N = number of participants; AoA = age of acquisition; IELTS = International English Language Testing Scores

The students' English Language test scores as required for admission onto their university course were converted to IELTS standard scores using the University of Nottingham English language qualification equivalencies. All participants spoke Malaysian English and Malay as their L2, and learnt both languages from a young age (range: 0–7 years; all except one reported 5 and below for age of Malaysian English acquisition). All participants had learnt the two languages for at least 11 years through formal education and were pursuing their tertiary education in English during the study. 22 participants also spoke at least one other Chinese language (generally Hokkien, Cantonese and/or Hakka). In addition, 3 participants reported to have learned a foreign language (Japanese or German) at a low proficiency (mean proficiency score < 2.2 from the scale of 7).

Participants were assigned randomly to either the low or high adverse condition groups. Between-subjects t-tests were then conducted to compare their linguistic experience and ability, so that the groups were matched in terms of their self-rated language proficiency, AoA and IELTS score.

The mean self-rated English and Mandarin proficiency did not differ between the two groups. Participants also did not differ in terms of their age of English acquisition (AoA) and IELTS standardized test scores.

#### Design and materials

The stimuli consisted of three groups of English minimal pairs: sixteen /t/-/d/ minimal pairs of which eight differed at the initial position (e.g., *tame–dame*) and eight at the final position (e.g., *sat–sad*), and sixteen /ε/-/æ/ minimal pairs (e.g., *leg–lag*). These two phonemic contrasts were chosen because they are difficult for the L1 Mandarin speakers (/t/-/d/ at word final position [5, 6] and /ε/-/æ/ [46]). The sixty-four English words consisted of nouns and verbs (complete list of stimuli can be found in the Supporting Information). The minimal pairs across the three groups were similar in word frequency (/t/-/d/ initial: 117.72, /t/-/d/ final: 101.32, /ε/-/æ/: 172.34 occurrences per million based on SUBTLEX-US [47]), number of syllables (/t/-/d/ initial: 1.0, /t/-/d/ final: 1.0, /ε/-/æ/: 1.3) and number of phonemes (/t/-/d/ initial: 3.38, /t/-/d/ final: 3.25, /ε/-/æ/: 3.5). The /t/-/d/ final minimal pairs were shorter than the other minimal pair groups in terms of the number of letters (/t/-/d/: 3.75, /t/-/d/: 3.50, /ε/-/æ/: 4.09, *F*(2,61) = 3.84, *p* < .05). For the training sessions, the sixteen /ε/-/æ/ minimal pairs were split into two sets of stimuli (trained and untrained). Half of the participants were trained with one set of stimuli, whereas the other participants were trained with the other set. Four speakers (2 females) with different English accents (female British English, male Southern Irish English, female American English, and male Irish English) recorded the spoken word stimuli. The stimuli were recorded in an Anechoic chamber using an AKG Perception 400 microphone connected to a Presonus FireBox, which was linked to an Apple Macbook Pro. Speech was recorded at 44.1.kHz (16 bit) using Amadeus Pro (version 2). Recordings were edited using Amadeus Pro and the volume of the recordings were normalised by amplifying the sound recordings of each speaker to an average root mean square (RMS) power of -25 dB (200 ms window). Stimuli spoken by the male Southern Irish English speaker were presented in the first training session, stimuli spoken by the female American English speaker were used in the second training session, and the stimuli spoken by the male Irish English speaker were presented in the third training session. The stimuli spoken by the female British English speaker were used in the pre- and post-tests.

The background noise consisted of 6-talker babble and was created by combining the audio recording of 6 native English speakers (3 females) taken from six BBC Radio 4 interviews in which the interviewees talked about their life and work. The interviewer's voice was edited out and the volume of each speaker was normalised using Amadeus Pro by amplifying the sound to an average root mean square (RMS) power of -25 dB (200 ms window). The resulting 6 audio files were combined into a single 6-talker babble mono audio file (44.1 kHz, 16 bit) of 6 minutes.

During pre-test, post-test and training with a high level of background babble, the multi-talker babble was played continuously during the task at half the stimulus level with a mean signal-to-noise ratio (SNR) of -2.6 dB (range -8.4 to 1.9 dB). During training with a low level of background babble, the multi-talker babble was played at one-tenth of the stimulus level with a mean SNR of 11.3 dB (range 5.6 to 15.9 dB). Praat [48] was used to obtain the RMS of the audio files in order to calculate the SNR. The experiment was approved by the University of Nottingham Malaysia Campus Research Ethics committee.

#### Procedure

All participants completed five sessions, one session per day. On the first day, participants completed the pre-test, followed by three training sessions spread across the following three consecutive days and then on the final day the participants completed the post-test. A 14-inch laptop (HP EliteBook 8460p) was used to run the training program and a mouse was used to record responses.

The pre- and post-tests consisted of two alternative forced-choice (2AFC) identification task. The stimuli (thirty-two minimal pairs: sixteen /ε/-/æ/ and sixteen /t/-/d/) in this task were repeated four times resulting in a total of a hundred and twenty-eight experimental trials. In each trial, the two words of a minimal pair were visually presented side by side at center of the computer screen and at the same time one of the words was presented auditorily. Participants were asked to indicate which word on the computer screen matched with the word they heard. Auditory stimuli were presented at a comfortable listening level set by each participant using Sony Headphones (MDR-NC8/WHI).

Participants completed eight practice trials in order to familiarize themselves with the task. Presentation of the minimal pairs was randomized. No feedback was provided after each trial. Only at the end of the practice trials and after each block of 32 experimental trials, the total percentage correct was presented. The pre- and post-tests each took approximately 15 minutes.

In the training sessions participants performed a Same-Different word discrimination task with background babble presented either at one-tenth or half of the stimulus level. Participants heard pairs of words and had to decide whether the words were the same or different by clicking on one of the two response buttons ("Same" or "Different") presented on the computer screen using a computer mouse. The second word was played 1000 ms after the first word. Participants received feedback after each response. After a correct response, the response button with the correct answer turned green and then the next trial started. After an incorrect response, the button with the incorrect answer turned red and the correct answer turned green. The word pair was played again before the next trial was presented and no response was needed.

Each training session lasted approximately 20 minutes. Participants were instructed to focus on the auditory words they heard and try to ignore the multi-talker babble played in the background. All participants gave written informed consent prior to the start of the experiment.

### Results and discussion

The mean percentage of correct identification was calculated for each phonemic contrast in the pre- and post-tests respectively (see Table 2). Data from the /t/-/d/ phonemic contrast at word initial position was excluded from the data analysis due to the near ceiling identification accuracy in pre-test for both participant groups (mean above 98%). Effect sizes (eta-squared, generalized eta-squared, Hedges’ *g*_*av*_ and Hedges’ *g*_*s*_) were calculated for significant findings using the spreadsheet provided by Lakens [49] and reported in the results sections of this and the following experiments. *F* and *p* values are only reported for significant effects.

**Table 2:** Mean percentage of correct identification in pre- and post-tests for each phonemic contrast and fixed level of adverse conditions (low and high level of background multi-talker babble) with standard error in parentheses.

	/ε/-/æ/		/t/-/d/ final	
Adverse Conditions	pre-test	post-test	pre-test	post-test
Low	73.1 (2.1)	77.2 (3.3)	73.7 (3.9)	75.0 (3.1)
High	72.1 (3.2)	78.2 (3.6)	72.3 (3.6)	72.8 (2.9)

The impact of training in adverse conditions with a low and high fixed level of background babble was examined in a 2 x 2 x 2 mixed ANOVA with the level of background multi-talker babble (low vs. high SNR) as the between-subject factor, and time of test (pre-test vs. post-test) and contrasts (/t/-/d/ final vs. /ε/-/æ/) as the within-subject factors. Overall, participants' perceptual performance was 3.0% better in the post-test (M = 75.8%, SE = 1.80) than in the pre-test (M = 72.8%, SE = 1.96), *F*(1,26) = 10.42, *p* < .01, η^2^_p_ = 0.29, 95% CI [1.09, 4.93], η^2^_G_ = 0.02. There was no interaction between the level of background babble and the time of test, indicating that both levels of background babble yielded similar levels of improvement in identification accuracy. Furthermore, there was no interaction between type of contrast and the time of test, which indicates that the training generalized to the stimuli of the untrained contrast /t/-/d/ final.

The above analysis included trained and untrained stimuli from the /ε/-/æ/ contrast. To assess whether the effect of the trained /ε/-/æ/ stimuli also generalized to untrained /ε/-/æ/ stimuli another mixed ANOVA was conducted (means are presented in Table 3). Results revealed that, as expected, the accuracy in the post-test (M = 77.7%, SE = 2.40) was significantly higher than in the pre-test (M = 72.6%, SE = 1.89), *F*(1,26) = 8.91, *p* < .01, η^2^_p_ = 0.26, 95% CI[1.60, 8.67], η^2^_G_ = 0.04 (5.1% difference). Importantly, there was no interaction between stimulus set (trained vs. untrained /ε/-/æ/) and the time of test, which indicates that the perceptual improvements generalized to the untrained stimuli.

**Table 3:** Mean percentage of correct identification in pre- and post-tests for each stimulus set and level of background multi-talker babble (with standard error in parentheses).

	Trained /ε/-/æ/		Untrained /ε/-/æ/	
Adverse Conditions	pre-test	post-test	pre-test	post-test
Low	74.3 (2.6)	78.8 (3.3)	71.9 (3.2)	75.7 (3.9)
High	71.7 (2.9)	79.5 (3.6)	72.5 (4.0)	77.0 (3.9)

HVPT training with a fixed level of background multi-talker babble successfully improved participants’ perceptual performance after a total of just one hour of training. Although participants identified all stimuli with a considerably high accuracy in the pre-test (mean 72.8%), the HVPT training in adverse conditions was able to further improve their overall perceptual performance by 3.0%.

Similar to findings from other training studies [1, 2, 24], identification accuracy improvements generalized to the untrained/novel words in post-test. Importantly, improvement was also generalized to stimuli of the untrained contrast /t/-/d/ final (see general discussion for further discussion). As far as we are aware, there is no other HVPT study that has reported generalization of training effects across phonemic contrasts, except for the study by Callan et al. [2]. Their training with the English /r/-/l/ contrast using HVPT benefited identification accuracy of the /b/-/v/ contrast as well.

The current findings also indicate that learning from the training transferred to a different speaker because the speaker of the post-test spoke a different variety of English (i.e., British English). Participants’ performance improved after training regardless of the level of adverse conditions used during training. This suggests that training in adverse conditions improves perceptual performance of non-native speakers and increasing the level of adverse conditions does not seem to influence the training outcomes. However, it is important to note that there were large individual differences in the current study. The percentage identification accuracy in the pre-test varied between 56% to 93%. In this experiment, the level of background multi-talker babble was not adapted to the participants’ individual performance level. Therefore, the participants' auditory system might not have been stressed sufficiently to maximize training benefits.

## Experiment 2

In our second experiment, we examined the impact of HVPT training in adaptive adverse conditions (HVPT-AAC). The level of background multi-talker babble (i.e., SNR) was determined before each training session using an adaptive staircase procedure. This individually determined SNR was then used in the subsequent training session with stimuli of the same speaker as used in the adaptive staircase procedure.

An adaptive staircase procedure is a psychometric method used to measure a person’s sensory capabilities and is used to determine the person’s threshold or limit to detect and discriminate similar and confusable physical stimuli [50]. This procedure has been used in a small number of phonetic training studies using acoustically manipulated synthetic stimuli [12, 51]. The acoustic properties of stimuli in these studies were carefully manipulated to produce speech stimuli that systematically differed from each other. As far as we are aware, no natural speech training studies have used an adaptive staircase procedure in a similar way because it would affect the naturalness and variability of the training materials. The adaptive staircase procedure in our HVPT-AAC, however, manipulates the volume of the background multi-talker babble (and thus the SNR) to increase or decrease phonemic discrimination difficulty. Thus, this does not affect the naturalness of the speech stimuli.

By combining the strengths of existing training methods and individualized levels of adverse conditions, HVPT-AAC is expected to be more effective than HVPT with a fixed level of background multi-talker babble (as used in Experiment 1) in terms of its training improvements and generalizability. Participants in Experiment 2 had more diverse Malaysian English proficiency levels (L1 and L2 Malaysian English speakers) to investigate whether the perceptual performance improvements were modulated by Malaysian English proficiency. It was expected that the training would benefit all Malaysian speakers, irrespective to their English proficiency level due to the adaptiveness of the adverse conditions in HVPT-AAC.

### Method

#### Participants

A group of 40 Malaysian participants (aged 16–23; 23 females) were recruited from the University of Nottingham, Malaysia campus. One participant was excluded due to the loss of a data file. Participants either spoke Mandarin (*n = 14*), Malay (*n = 14*) or Malaysian English (*n = 11*) as their L1. All participants also spoke both Malaysian English and Malay from young age (from 0–7 years old). One L1 Malay speaker was also able to speak Mandarin, but at a very low proficiency level. All participants had normal or corrected to normal vision, and had no history of any hearing, speech and reading disorders. The language background of the participants was assessed with the same language questionnaire as used in Experiment 1 (see Table 4).

**Table 4:** Participants’ demography, mean self-rated language proficiency across the three languages and English language test scores, according to their L1.

L1	N	Age	Self-rated English Proficiency	Self-rated Mandarin Proficiency	Self-rated Malay Proficiency	AoA	IELTS
Mandarin	14	18.64	4.96	5.87	4.87	3.86	7.04
Malay	14	18.00	5.23	1.50	6.75	5.14	6.82
Malaysian English	11	17.81	6.23	3.34	5.89	2.18	7.32

*Note*. L1 = first language; N = number of participants; AoA = age of acquisition; IELTS = International English Language Testing Scores

To examine whether the language groups differed in terms of their English language proficiency, a between-subjects one-way ANOVA was conducted with IELTS scores and the mean self-rated English proficiency. Results revealed that the groups differed significantly in terms of self-rated English proficiency, *F*(2,38) = 9.47, *p* < .001 and IELTS scores, *F*(2,38) = 3.57, *p* < .05. However, post-hoc t-tests with Bonferroni correction indicated that L1 Malay and L1 Mandarin participants did not differ in terms of their self-rated English proficiency scores and IELTS sores. For IELTS scores, only L1 Malay participants were found to score significantly lower compared to L1 Malaysian English participants (*p* < .05). As expected, L1 Malaysian English participants reported a higher level of English proficiency compared to the Malay and Mandarin groups (*p*s < .01).

#### Design and stimuli

The design and stimuli were identical to Experiment 1. The two phonemic contrasts used in the experiment are also difficult for the L1 Malay and Malaysian English speakers (absence of voiced stop consonant including /d/ at word final position in the Malay language except for borrowed words [52, 53]; ambiguous representations of /ε/-/æ/ among L1 Malay [54] and Malaysian English speakers [55]). During the pre-test and post-test, the multi-talker babble was played continuously during the tasks at half of the target stimulus volume level with a mean signal-to-noise ratio (SNR) of -2.6 dB while the target stimuli were presented.

#### Procedure

The procedures of the pre-test, training task and post-test were identical to Experiment 1. The difference with Experiment 1 was the additional adaptive staircase procedure, which took about 5 minutes and was completed before each of the training sessions.

The adaptive staircase procedure was used to determine the optimal volume level of background multi-talker babble that would be used for the subsequent training session. Participants performed a Same-Different word discrimination task similar to the training task in Experiment 1. However, the volume level of the background multi-talker babble was manipulated based on the accuracy of the preceding response. A three-down, one-up adaptive staircase algorithm was used so that the participant reached an accuracy level of 79.4% [50].

The procedure adjusted the volume of the multi-talker babble within a range from 0 to 100, where 0 indicates that no multi-talker babble is presented and 100 refers to an identical volume of the babble (noise) and the target words (signal), the mean SNR of the maximum volume level (100) was -8.7 dB. In the first trial, the volume of the multi-talker babble was set to 0 and the amount of change (step size) was set initially at 64. For every reversal, the step size was divided by 2 so that the amount of change in volume decreased across trials. A reversal was defined as a correct response followed by an incorrect response or vice versa. If the response in a trial was correct, the volume was increased by 1 x the step size, if it was incorrect, it was decreased by 3 x the step size. For instance, the multi-talker babble volume increased from 0 to 64 (SNR = -4.8 dB) after the first correct trial. In the second trial, the volume was increased by another 64 but capped at 100 (SNR = -8.7 dB) when the response was correct again. However, if the response was incorrect in the second trial, the volume decreased by 96 (3 x 32 steps; step size was reduced by half from 64 to 32 due to the first reversal which took place when a correct response was made in first trial followed by an incorrect response made in second trial). Thus, the volume was reduced from 64 to 0 because the minimum volume level was 0. The minimum step size was 1. The staircase procedure stopped after 12 reversals. At the end of the staircase procedure the final level of background multi-talker babble (threshold level) was set at the average of the volumes from the last 4 reversals. The adaptive staircase procedure used stimuli from the same speaker that was used in the following training session. Importantly, no feedback was provided in the adaptive staircase procedure.

As in Experiment 1, participants performed the Same-Different discrimination task with feedback during the training. The only difference with Experiment 1 was that the background multi-talker babble was set at the level determined by the adaptive threshold procedure before the training session.

### Results and discussion

Similar to Experiment 1, pre-test performance differed widely between participants (accuracy range from 53% to 94%). The adverse levels used during training (SNR) were calculated across participants for each speaker and a one-way repeated measure ANOVA was conducted to compare the SNR levels between speakers. Results showed that the male Southern Irish English speaker was least intelligible (SNR range -8.8 to 31.2 dB, mean SNR = 2.5 dB, SE = 1.3) compared to the other male Irish English speaker (SNR range -8.5 to 17.3 dB, mean SNR = -2.7 dB, SE = 0.85) and the female American English speaker (SNR range -7.9 to 4.2 dB, mean SNR = -3.4 dB, SE = 0.43), *F*(2,80) = 51.42, *p* < .001.

The mean accuracies of the pre- and post-tests are presented in Table 5. The data from the /t/-/d/ phonemic contrast at word initial position was again excluded from the data analysis due to the near ceiling identification accuracy in pre-test for all L1 groups (mean above 95%).

**Table 5:** Mean correct identification percentage in pre- and post-tests for each phonemic contrast and first language (L1) group (with standard error in parentheses).

	/ε/-/æ/		/t/-/d/ final	
L1	pre-test	post-test	pre-test	post-test
Mandarin	70.9 (2.8)	81.2 (2.0)	71.7 (3.3)	80.4 (3.5)
Malay	80.3 (2.2)	88.8 (1.8)	72.3 (3.5)	79.91 (3.8)
Malaysian English	81.5 (4.0)	86.4 (3.7)	87.2 (2.7)	88.6 (2.8)

The participants' identification accuracy before and after training was examined in a 3 x 2 x 2 mixed ANOVA with time of test (pre- vs. post-test) and phonemic contrast (/ε/-/æ/ vs. /t/-/d/ final) as the within-subject factors and L1 (Mandarin vs. Malay vs. Malaysian English) as the between-subject factor.

Overall perceptual performance improved significantly after training (pre-test: M = 76.8%, SE = 1.69; post-test: M = 84.0%, SE = 1.38), *F*(1,37) = 42.50, *p* < .001, η^2^_p_ = 0.54, 95% CI[4.77, 9.07], η^2^_G_ = 0.10. A main effect of L1 was found, *F*(1,26) = 4.42, *p* < .05, η^2^_p_ = 0.20, 95% CI, η^2^_G_ = 0.12. Tukey’s HSD post-hoc tests showed a significant lower identification accuracy for the Mandarin group (M = 76.0%, SE = 2.01) relative to the Malaysian English group (M = 85.9%, SE = 2.72), *p* < .05. Furthermore, a marginally significant interaction was observed between time of test and L1, *F*(2,37) = 3.12, *p* = .06, η^2^_p_ = 0.15, 95% CI, η^2^_G_ = 0.08, indicating that improvements observed potentially differed between the language groups. Post-hoc paired-samples t-tests with Bonferroni correction revealed that Mandarin, *t*(13) = 4.56, *p* = .001, *g*_av_ = 1.03 and Malay, *t*(13) = 4.53, *p* < .001, *g*_av_ = 0.82 groups improved significantly in post-test, whereas Malaysian English group did not. Identification accuracy for stimuli of the /ε/-/æ/ and the /t/-/d/ final contrasts improved similarly because no interaction was found between phonemic contrast and the time of test. Furthermore, there was no three-way interaction between time of test, L1 and phonemic contrast and no other significant effect were found.

Similar to Experiment 1, training generalization from trained to untrained /ε/-/æ/ stimuli was examined in a 3 x 2 x 2 mixed ANOVA (see Table 6 for means).

**Table 6:** Mean percentage of correct identification in pre- and post-tests for each stimulus set and L1 group (with standard error in parentheses).

	Trained /ε/-/æ/		Untrained /ε/-/æ/	
L1	pre-test	post-test	pre-test	post-test
Mandarin	70.8 (2.6)	82.1 (2.3)	71.0 (4.1)	80.4 (2.6)
Malay	80.6 (2.9)	90.2 (1.9)	79.9 (2.8)	87.5 (2.5)
Malaysian English	84.7 (4.8)	86.1 (4.1)	78.4 (3.7)	86.6 (4.0)

The analysis revealed again a higher identification accuracy in post-test (M = 85.4%, SE = 1.49) compared to pre-test (M = 77.2%, SE = 1.83), *F*(1,36) = 46.20, *p* < .01, η^2^_p_ = 0.56, 95% CI[5.57, 10.30], η^2^_G_ = 0.11. Importantly, no interaction was found between stimuli set and time of test, indicating that perceptual improvements were the same for trained and untrained stimuli. Again, a main effect of L1 was found, *F*(2,36) = 3.65, *p* < .05, η^2^_p_ = 0.17, η^2^_G_ = 0.11. Tukey’s HSD post-hoc tests revealed that overall identification accuracy of the Mandarin group (M = 76.06%, SE = 2.22) was lower than the Malay group (M = 84.74%, SE = 1.69), *p* < .05, but only marginally different from the Malaysian English group (M = 83.95%, SE = 3.70), *p* = .10. No interaction was found between L1 and time of test, although numerically there were differences between the groups in term of their improvements from pre- to post-test (L1 Mandarin: 10.4%, L1 Malay: 8.8%, L1 Malaysian English: 4.5%).

The HVPT-AAC improved the participants’ perceptual performance by 7% after a total of just one hour of training. The improvements were modulated by English proficiency because larger improvements were found for L1 Mandarin (9.5%) and L1 Malay speakers (8.1%) relative to L1 Malaysian English speakers (3.1%) whose English proficiency was higher than the other two groups. Importantly, the training improvement across groups generalized from trained to untrained /ε/-/æ/ stimuli and to stimuli of the untrained /t/-/d/ final contrast.

#### HVPT in fixed vs. adaptive levels of adverse conditions

To compare the effectiveness of HVPT in fixed adverse conditions (Experiment 1) with HVPT-AAC (Experiment 2), the identification accuracy of the L1 Mandarin speakers from Experiment 1 and 2 were analyzed together. Data from the groups with low and high levels of background multi-talker babble of Experiment 1 were collapsed because there was no significant difference in training between these two conditions. To ensure that training effects of both experiments were not affected by differences between the participant groups, the linguistic background and pre-test performance of the participants were compared between Experiment 1 and 2. Between-subject t-tests confirmed that participants of both experiments did not differ in terms of their self-rated English proficiency, self-rated Mandarin proficiency, IELTS standard scores, AOA, and most importantly, their pre-test identification accuracy.

**Fig 1:**
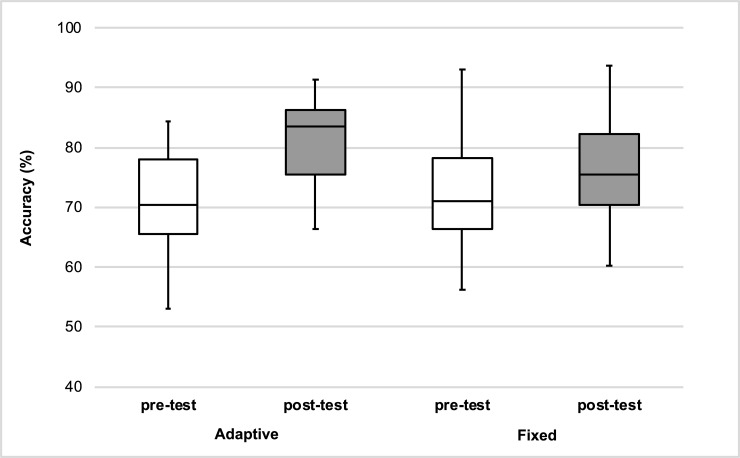
Interaction between type of adverse conditions and time of test. Box-plots of pre-tests and post-tests accuracies for HVPT in fixed adverse conditions and HVPT in adaptive adverse conditions (HVPT-AAC).

The mean percentage of correct identification between Experiment 1 and 2 was examined in a 2 x 2 x 2 mixed ANOVA with type of adverse conditions (fixed vs. adaptive) as the between-subject factor, and time of test (pre-test vs. post-test) and phonemic contrast (/t/-/d/ final vs. /ε/-/æ/) as the within-subject factors. The analysis revealed improved perceptual performance in post-test (M = 77.5%, SE = 1.39) compared to pre-test (M = 72.3%, SE = 1.52), *F*(1,40) = 41.07, *p* < .01, η^2^_p_ = 0.51, 95% CI[4.30, 8.26], η^2^_G_ = 0.001. Importantly, an interaction was found between time of test and type of adverse conditions, *F*(1,40) = 11.11, *p* < .01, η^2^_p_ = 0.22, η^2^_G_ = 0.02 (see Fig 1). Post-hoc paired-samples t-tests with Bonferroni correction revealed significant identification improvement in the HVPT-AAC, *t*(13) 4.56, *p* = .001, *g*_*av*_ = 1.03 and the fixed level of adverse condition, *t*(27) = 3.28, *p* < .01, *g*_*av*_ = 0.28. An additional between-subjects t-test was conducted to further examine the interaction. The t-test comparing the improvement size (difference in identification accuracy between pre- and post-test) between the two training conditions showed greater training benefits for HVPT-AAC (10%) compared to HVPT with a fixed level of adverse conditions (3%), *t*(18.17) = 2.86, *p* < .01, *g*_*s*_ = 0.92. Thus, the adaptive nature of the adverse conditions during training enhanced the effectiveness of HVPT. An important question is whether the training results were long-lasting. This was investigated in the Experiment 3.

## Experiment 3

Numerous training studies have shown that perceptual training results in long-term benefits [1,15,17,19] and that it can serve as an indication of robust L2 category formation [15] or an effective perceptual strategy learnt to perceive L2 speech sounds in other settings. This retention benefit is important because it would suggest that phonetic training can be used to resolve L2 speakers’ difficulties in perceiving non-native phonetic contrasts. To investigate the long-term effects of HVPT in adverse conditions, an additional post-test was conducted with the L1 Mandarin participants of Experiments 1 and 2, 6 months after the last testing day.

### Method

#### Participants

All fourteen L1 Mandarin speaking participants from Experiment 1 (aged 18–23; 7 females) and thirteen (7 from low babble, 6 from high babble) out of the twenty-eight L1 Mandarin speaking participants from Experiment 2 (aged 18–22; 9 females) returned to complete a second post-test 6 months after the last testing session.

The linguistic background of these two groups of participants (see Table 7) was compared to ensure that the groups were matched. The mean self-rated English and Mandarin proficiency and the IELTS standardized test scores did not differ between the two groups. However, the English AoA of the adaptive adverse condition participants was significantly later than the fixed level adverse condition participants, *t*(25) = 2.17, *p* < .05, *g*_*s*_ = 0.8. This AoA difference was unexpected and likely due to a difference in instruction between the experiments and the subsequent interpretation of the term in the questionnaire by the participants. Participants in Experiment 1 interpreted the term as the age first exposed to the language through listening, whereas participants in Experiment 2 interpreted the term as the age first starting to learn speaking English.

**Table 7:** Participants’ demography, mean self-rated language proficiency in each language and their English language test scores.

Adverse Conditions	N	Age	Self-rated English Proficiency	Self-rated Mandarin Proficiency	AoA	IELTS
Fixed	14	18.64	4.56	5.54	2.00	7.31
Adaptive	13	18.69	4.87	5.87	3.93	7.04

*Note*. N = number of participants; AoA = age of acquisition; IELTS = International English Language Testing Scores

#### Design and stimuli

The stimuli, design and procedure were identical to the pre- and post-tests used in Experiments 1 and 2.

### Results and discussion

**Fig 2:**
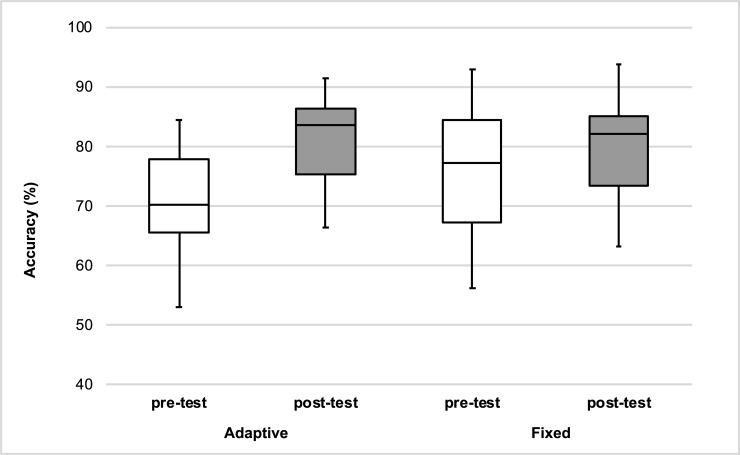
Interaction between time of test and type of adverse conditions observed with L1 Mandarin speakers. Box-plots of pre-tests and post-tests accuracies for HVPT in fixed adverse conditions and HVPT in adaptive adverse conditions (HVPT-AAC).

The mean identification accuracies of the delayed post-test after 6 months are presented in Table 8 together with means of the pre-test and immediate post-test. The first mixed ANOVA analysis investigated whether the training data of the participants included in this follow up experiment replicated the findings reported earlier. The results were similar to those reported in the combined analyses of Experiment 1 and 2 (see Experiment 2). Identification accuracy in the post-test (M = 79.9%, SE = 1.7) was higher than pre-test (M = 73.6%, SE = 2.27), *F*(1,25) = 29.30, *p* < .01, η^2^_p_ = 0.54, 95% CI[5.22, 8.68], η^2^_G_ = 0.08. Furthermore, the interaction between time of test and type of adverse conditions revealed again that training in adaptive adverse conditions was more effective than training in a fixed level of adverse conditions, *F*(1,25) = 5.31, *p* < .05, η^2^_p_ = 0.18, η^2^_G_ = 0.02 (see Fig 2). Post-hoc paired-samples t-tests with Bonferroni correction revealed significantly higher identification accuracy in post-test compared to pre-test for both conditions, *t*s > 3.14, *p*s < .01, *g*_*av*_ > .26. In order to further explore the interaction, an additional between-subject t-test was conducted to compare the improvement size yielded by both training conditions. The t-test revealed a larger improvement for HVPT in adaptive adverse conditions (M = 9.54%, SE = 2.09) compared HVPT with a fixed level of adverse conditions (M = 3.85%, SE = 1.23), *t*(20.82) = 2.35, *p* < .05, *g*_*s*_ = 0.86.

**Table 8:** Mean percentage of correct identification in pre-, post- and 6-months delayed post-test for each phonemic contrast and HVPT training in fixed and adaptive adverse conditions (with standard error in parentheses).

	/ε/-/æ/			/t/-/d/ final		
Adverse Conditions	pre-test	post-test	6-months delayed post-test	pre-test	post-test	6-months delayed post-test
Fixed	77.4 (2.8)	83.2 (3.6)	82.3 (3.8)	74.3 (3.9)	76.2 (3.1)	76.2 (4.5)
Adaptive	70.9 (2.8)	81.2 (2.0)	81.7 (2.8)	71.6 (3.3)	80.4 (3.5)	77.0 (3.1)

To examine retention of the training improvements after 6 months, a 2 x 2 x 2 mixed ANOVA with time of test (post-test vs. 6-months delayed post-test) and phonemic contrast (/ε/-/æ/ vs. /t/-/d/ final) as the within-subject factors, and level of adverse conditions (adaptive vs. fixed) as the between-subject factor. This analysis revealed no significant effects or interactions, except for the marginal effect of phonemic contrast, *F*(1,25) = 3.80, *p* = .06, η^2^_p_ = 0.13, 95% CI[0.26, 9.61], η^2^_G_ = 0.04. Thus, overall identification performance did not significantly reduce 6 months after the immediate post-test.

**Fig 3:**
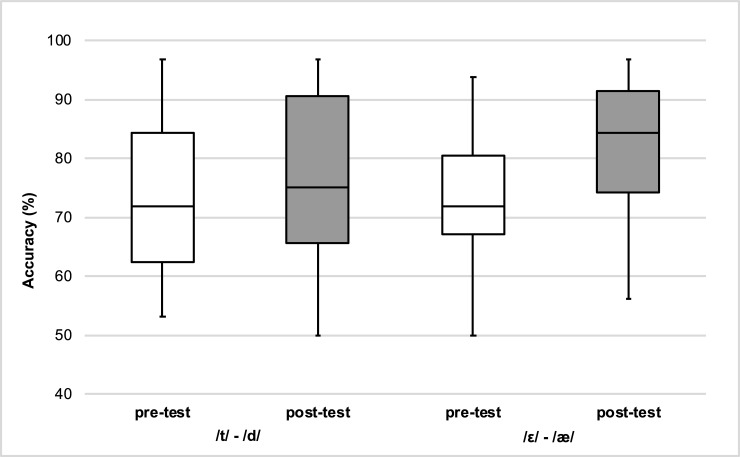
Interaction between time of test and phonemic contrast observed with L1 Mandarin speakers. Box-plots of pre-tests and 6-months delayed post-tests accuracies for the /t/-/d/ and /ε/-/æ/ phonemic contrasts.

Next, the identification accuracy in the pre-test and the 6-months delayed post-test were examined. This analysis revealed again that perceptual performance in the 6-months delayed post-test (M = 79.2%, SE = 2.28) was higher than in the pre-test, *F*(1,25) = 11.30, *p* < .01, η^2^_p_ = 0.31, 95% CI[2.23, 9.29], η^2^_G_ = 0.05. There was a trend towards an interaction between time of test and phonemic contrast, *F*(1,25) = 3.25, *p* = .08, η^2^_p_ = 0.11, η^2^_G_ = 0.01, which could be driven by the difference in improvements achieved between the /ε/-/æ/ and /t/-/d/ final phonemic contrasts (see Fig 3). Post-hoc paired-sample t-tests with Bonferroni correction revealed that identification accuracy of /ε/-/æ/ stimuli in the 6-months delayed post-test (M = 82.0%, SE = 2.29) was higher than in the pre-test (M = 74.0%, SE = 2.05), *t*(26) = 4.76, *p* < .01, *g*_*av*_ = 0.38. However, no significant difference in identification accuracy for untrained /t/-/d/ stimuli was found between pre-test (M = 72.9%, SE = 2.49) and at the 6-months delayed post-test (M = 76.6%, SE = 2.65).

The generalization of the training effects from trained to untrained /ε/-/æ/ stimuli were examined using the data of the pre-test and the 6-months delayed post-test. A mixed ANOVA (see Table 9) revealed, as expected, that the identification accuracy of the /ε/-/æ/ stimuli at the 6-months delayed post-test was higher than at pre-test, *F*(1,25) = 24.04, *p* < .001, η^2^_p_ = 0.49, 95% CI[4.57, 11.19], η^2^_G_ = 0.09. There was a trend towards an interaction between the time of test and the type of adverse conditions, *F*(1,25) = 3.37, *p* = .08, η^2^_p_ = 0.12, η^2^_G_ = 0.01. Additional between-subjects t-test revealed that after 6 months the perceptual improvements with training in adaptive adverse conditions (M = 10.8%, SE = 2.40) was only marginally larger than training in a fixed level of adverse conditions (M = 4.9%, SE = 2.10), *t*(25) = 1.84, *p* = .08, *g*_*s*_ = 0.68. No other effects or interactions were found, which suggests that after six months the training benefits still generalized from trained to untrained /ε/-/æ/ stimuli.

**Table 9:** Mean percentage of correct identification percentage in pre-, post- and six months post-tests for each stimulus set and HVPT training in fixed or adaptive adverse conditions (with standard error in parentheses).

	Trained /ε/-/æ/			Untrained /ε/-/æ/		
Adverse Conditions	pre-test	post-test	6-months delayed post-test	pre-test	post-test	6-months delayed post-test
Fixed	76.9 (3.1)	83.9 (3.6)	84.6 (4.3)	77.9 (3.3)	82.5 (4.1)	80.0 (4.0)
Adaptive	70.8 (2.6)	82.1 (2.3)	79.9 (2.9)	71.0 (4.1)	80.4 (2.6)	83.5 (3.2)

The delayed post-test revealed that perceptual improvements were retained 6 months after training as participants performed similarly in the delayed post-test as in the immediate post-test. The analysis that compared the data of the pre-test with the delayed post-test after 6 months still showed significant perceptual improvements, which was mainly driven by the /ε/-/æ/ stimuli. Further analyses revealed that relative to the pre-test, the delayed post-test after 6 months again showed evidence of generalization from trained to untrained /ε/-/æ/ stimuli. Importantly, there was some evidence that the retained training effect on /ε/-/æ/ stimuli was larger for training in adaptive adverse conditions than training in a fixed level of adverse conditions.

## General discussion

The present experiments revealed that after a total of just one hour of high variable phonetic training with English phonemic contrasts in adverse listening conditions, the perceptual performance of Malaysian multilinguals significantly improved. Moreover, the perceptual improvements were successfully retained six months after training, demonstrating sustainability of this rapid and effective training paradigm. Consistent with previous findings, the training benefits generalized to novel stimuli of the trained English contrast, /ε/-/æ/ and to novel speakers [1, 3, 6, 44]. Furthermore, HVPT-AAC generalized to untrained /t/-/d/ final stimuli. This cross phonemic-contrast generalization can be explained by the findings of Flege [6], who demonstrated that when the final closure voicing and released burst cues were removed (which is common in conversational speech), the acoustic information retained in preceding vowel (e.g. spectral quality, duration and onset/offset of formants) was sufficient to cue voicing judgment for the following English /t/-/d/ stop consonants, for the native English speakers as well as the Chinese speakers after training. Therefore, the cross phonemic-contrast learning may be attributed to the successful selective attention shift to the word middle vowel position and away from the background noise, instead of mastering the phonetical differences between the untrained /t/-/d/ stop stimuli.

Before discussing the implications of the present findings, it is important to note that the moderate to high English proficiency of the current participants presents actually a challenge for the evaluation of HVPT-AAC because the performance of some participants was already close to the ceiling level in the pre-test. Thus, any training improvements observed with HVPT-AAC are therefore likely to be an underestimation of the impact that the training might have with less proficient participants.

Similar to previous training studies that compared different training paradigms or training effects between different populations [3, 4, 18, 19, 23, 24, 44, 56], our experiments did not include control groups that did not do any training. Repeated exposure to the same stimuli spoken by the British English speaker in pre- and post-tests may potentially contribute to participants’ better performance in the post-test because they could attune to the idiosyncratic cues associated with that speaker [57]. However, most training studies involving a control group found that it is difficult for participants to learn through mere exposure in pre- and post-tests [6, 16, 17, 21, 58, 59]; except for one study [51] in which participants were exposed repetitively and intensively to only two synthetic *rock-lock* and *road-load* continuums. Therefore, phonetic learning might have been more straightforward than in other training studies that involved high variability training stimuli. Nevertheless, we do not rule out the possibility that the perceptual improvement might have induced some learning through repetitive exposure to the stimuli and/or background babble.

Importantly, we found greater benefits of HVPT-AAC (Experiment 2) when the adverse conditions level was adapted for each participant than of HVPT with fixed adverse conditions (Experiment 1). The benefits of HVPT-AAC can be attributed to its adaptive ability to individualize the training level to participants with varied pre-test performance, as well as to the training stimuli used in each training session/day that were produced by different speakers. As reported in Experiment 2, we found that the intelligibility of the three speakers used during training differed. This is consistent with the findings by Peng and Wang [31] who showed that background noise impacts English accents and individuals differently. The adaptive feature of HVPT-AAC also accounted for the different impact of background noise on the accented speech through the establishment for each participant of an individualized background noise level for each talker (before each training session) so that the participant was able to reach an overall accuracy level of 79.4%.

The variability of perceptual training studies in the literature in terms of training methodologies (e.g., training hours, training tasks, stimulus types) makes comparisons across studies difficult. Some studies reported very large perceptual improvements after training but they usually involved a substantial amount of training time. For example, Richie and Kewley-Port [40] reported a 29% improvement in vowel perception after 6 hours of training although no significant improvements were found in word recognition. Nishi et al. [19] trained Japanese learners of English across 9 days and perceptual improvements were maximally 26% after training. However, the total training time in this study was 13.5 hours. Wang and Munro [17] used L1 Mandarin or Cantonese speakers and a similar training paradigm as in the current study but without adverse conditions. A 20% improvement was found that was retained after 3 months and the training effect also generalized to novel words produced by novel speakers. However, their training took up to 24 hours spread over 24 sessions.

**Fig 4:**
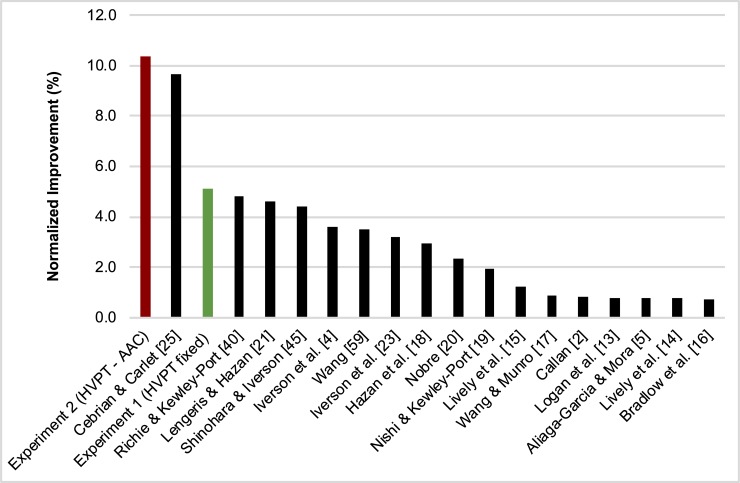
Normalized percentages of improvement. The normalized percentage of improvements in the current study and those reported in other studies. The green colored column refers to Experiment 1 (HVPT in fixed levels of adverse conditions; /e/-/a/ stimuli only), whereas the red colored column refers to the Mandarin speakers of Experiment 2 (HVPT in adaptive adverse conditions (HVPT-AAC); /e/-/a/ stimuli only). The normalized percentage was calculated by dividing the percentage of improvement after training by the number of training hours.

To be able to compare the perceptual improvements in our study to studies in the literature involving only non-native speakers and natural English speech stimuli, we calculated for the current and previous training studies the normalized percentage of improvements by dividing the percentage of perceptual improvement (largest percentage reported in each paper) by the number of training hours. The normalized percentages are presented in Fig 4.

As can be seen in Fig 4, the normalized percentage of improvement after HVPT-AAC is the largest (10.4%) and also the improvement of HVPT in a fixed level of adverse conditions (5.1%) is larger than most of the other training studies (mean of other studies: 2.7%). The only study that comes close to the level of normalized improvement of the HVPT-AAC is the study by Cebrian and Carlet [25]. The normalized improvement in Cebrian and Carlet's study (9.7%) was based on improvements in the detection of the /d/ consonant, which had a below chance identification accuracy in the pre-test (46%), Therefore, a substantial improvement is to be expected in the post-test. Note that the improvements obtained with our HVPT-AAC reported in Fig 4 (10.4% and 5.1%) were based on a vowel contrast. In Cebrian and Carlet's study, the vowel /i/ had the second highest training improvement, with a normalized improvement of only 2.7%. Overall, our HVPT-AAC seems to be more effective than other perceptual training paradigms reported in the literature. However, averaging the percentage improvements across training hours may not be the ideal normalization treatment for studies involving many hours of training because of the non-uniform rate of improvement across training. The largest improvement is usually achieved during the first few hours of training (e.g., [41]). However, the significant improvement observed for the HVPT-AAC in just one hour of training is still quite promising. The ultimate outcome of perceptual training would be to improve the non-native speakers’ perceptual performance to native-level performance levels. No training study has achieved this (e.g., [6, 16, 17, 43]). It is unclear yet how successful HVPT-AAC could be when it would be used for a longer time. Thus, future research should explore the effectiveness of HVPT-AAC across a larger number of training sessions.

The high variability training in HVPT-AAC has likely contributed to its effectiveness (see [1, 13–15]), because it was designed to maximize the variability in terms of the phonetic context in which the critical phoneme occurred (using different minimal pairs), number of speakers, gender of the speakers and the variety of English accents heard during the training. With disruptive effects of noise, non-native listeners are affected by talker variation (caused by acoustical variability produced by different talkers) due to the formation of ambiguous phonetic categories and their lack of exposure to the range of production variability [35]. Hence, the inclusion of training stimuli variability in terms of English variety would be particularly beneficial for non-native listeners, if not necessary, to teach listeners how individuals who speak different accented English vary in their word productions.

Another key factor of HVPT-AAC that has likely contributed to the effectiveness is the use of adverse conditions during training. The adverse conditions make it more difficult to identify the spoken words and stressed the speaker's auditory system so that more attention had to be used to detect relevant acoustic cues. Our results indicate that merely increasing the level of adverse conditions did not impact the perceptual improvements. Critically, it was the adaptive nature of the adverse conditions in HVPT-AAC, which were determined for each participant before each training session, that lead to the highest identification improvements. Unlike HVPT-AAC that involved multi-talker babble to degrade target speech, Iverson, Hazan and Bannister [4] altered the way non-native listeners weight the significance of different acoustical cues by enhancing the relevant acoustical cues to improve phonetic salience between the English phonemic contrast /r/-/l/. Although training with acoustical cues enhanced, it did not direct participants’ attention away from the less relevant secondary cues. Rather, it changed participants’ strategy of using the secondary cues (e.g., increasing response bias in choosing /l/ for short transitions and high F2 frequencies). Something similar could also have happened in the present study. However, it is not yet clear whether the participants’ perceptual improvement was driven by successful learning of attending to the acoustic cues in a similar way as the native speakers do.

As predicted by Best’s Perceptual Assimilation Model [7] and Kuhl’s Native Language Magnet Theory [8], the L1 Mandarin Malaysian speakers assimilated the British English /ε/-/æ/ phonemic sounds into a single category because of the absence of categorical distinction in Malaysian English [7, 56]. The low front vowel /æ/ was later identified as the more operative vowel representation as the two phonetic sounds were matched to the /æ/ phonemic words at a higher rate [55]. According to Flege’s Speech Learning Model [9], phonetic salience between phonemic contrasts are essential before distinct phonetic categories can be established for the sounds. It is thus important to improve speakers’ realizations of the two phonetically distinct English sounds to promote perceptual acuity. The discrimination task used in our training aimed to improve Malaysian English speakers’ discrimination sensitivity and the training impact was reflected in participants’ improvement in post-test identification accuracy. The perceptual improvement also suggest that phonetic salience could be learnt within an hour of phonetic training in adverse conditions.

A successful training paradigm should train participants to perceive in a more native-like manner by directing them to the critical acoustical cues the native speakers attend to. When trained participants learnt how to acquire sufficient acoustical information to cue accurate judgments, the reliance and interference of L1 could be reduced. Participants in the present experiments could have learnt to acquire acoustical information from the stimuli of different English varieties (Southern Irish English, American English and Irish English) and successfully transferred the learning to identify phonemic pairs of another English variety (British English). This type of learning transfer could indicate the effectiveness of HVPT-AAC by re-directing participants’ attention in perceptual tasks rather than by developing highly detailed representations of the trained speech materials.

In today’s society, English serves as a global lingua franca for communication in business and academic settings between speakers with different L1 backgrounds [59, 60]. In situations where individuals often need to travel and communicate with people from different cultures and language backgrounds who speak different variety of English (usually for education and business purposes), HVPT-AAC could be very beneficial to improve perceptual sensitivity towards any target language within a short amount of time. Another advantage of HVPT-AAC is its adaptability to learners with different proficiency levels and to different spoken material and speakers. Even the highly proficient L1 Malaysian English participants who already performed remarkably well in the pre-test benefitted from further training. Future research could, for example, examine the effectiveness of HVPT-AAC with other languages and examine how to further improve its effectiveness not only in speech perception but also in terms of improving speech production.

## Supporting information

S1 File Stimuli. English minimal pairs used in the experiments.
(DOCX)

S2 File Data. Participants' percentag of correct identifications for the conditions in each experiment.
(XLSX)
