# Supplementary material for: High variability phonetic training in adaptive adverse conditions is rapid, effective, and sustained
Source: PLoS One. 2018 Oct 9;13(10):e0204888. doi: 10.1371/journal.pone.0204888 (PMC6177151; doi:10.1371/journal.pone.0204888)
Supplement: S1 File — English minimal pairs used in the experiments. (DOCX) [file pone.0204888.s001.docx]

**S1 Stimuli.** English minimal pairs used in the experiments

**/t/-/d/ initial**: tame-dame, tart-dart, ties-dies, tin-din, tip-dip, town-down, tuck-duck, tug-dug.

**/t/-/d/ final:** bait-bade, bat-bad, beat-bead, bet-bed, bit-bid, rot-rod, sat-sad, white-wide.

**/ε/-/æ/:** head-had, men-man, mesh-mash, mess-mass, peck-pack, set-sat, shell-shall, send-sand, leg-lag, merry-marry, pen-pan, flesh-flash, pet-pat, lend-land, kettle-cattle, celery-salary.
